# Environmental exposure to agrochemicals and allergic diseases in preschool children in high grown tea plantations of Sri Lanka

**DOI:** 10.1186/s13223-018-0308-z

**Published:** 2018-12-04

**Authors:** Sanath Thushara Kudagammana, Keerthi Mohotti

**Affiliations:** 1 Department of Paediatrics, Faculty of Medicine, University of Peradeniya, Peradeniya, 20400 Sri Lanka; 2 Tea Research Institute, Thalawakelle, Sri Lanka

## Abstract

**Background:**

Exposure to agrochemicals is one of the many aetiological agents, postulated to cause allergic diseases. In this study, we have compared the prevalence of allergic diseases among preschool children growing in environments exposed to agrochemicals and artificial fertilizers with those who are not exposed to them.

**Methods:**

Our study was conducted on preschool children in two tea estates in the hill country of Sri Lanka, one using conventional agricultural practices and the other using organic practices. Data collection was done by using an interviewer administered questionnaire. Children with potential allergic conditions were further evaluated clinically by medical officers. Blood was drawn for full blood count and a blood picture.

**Results:**

Data from 81 preschool children from an organic estate (Haputale) and 101 preschool children from a conventional estate (Thalawakelle) were analysed. Wheezing was noted in 41.2% of children from the organic estate and 59.8% from the conventional estate. The respective percentages for allergic rhinitis were as 37.7% and 82.5% while for eczema they were 17.5% and 20.28%. Among the two populations percentages of eosinophilia > 600/mm^3^ were as 26.1% and 34.1% respectively.

**Conclusions:**

Allergic conditions were more common in preschool children with environmental exposure to agrochemicals and chemical fertilizers when compared to that of organic cultivation systems. Stricter rules are needed when using agrochemicals to prevent their harmful effects, including allergic diseases, on children.

## Background

With the increasing world population, there is immense pressure on the agricultural sector to increase the efficiency of food production from an available extent of land. This has led to the growing of newer species using genetic modification and to excessive use of potent agrochemicals and chemical fertilizers. Although there is no doubt that this has increased the crop yield, there is a growing concern regarding the unnecessary effects on the environment and the health of the world population. While the use of agrochemicals is well controlled in the developed world, this is not so in the developing world.

Allergic diseases are common during childhood. They include childhood asthma, allergic rhino conjunctivitis, eczema and food allergies. The causes of allergic disorders are numerous and include genetic and environmental factors. Studies have shown that prenatal exposure to viral infections is associated with allergic diseases during childhood [1]. Exposure to pesticides and weedicides and other environmental pollutants is another postulated group of aetiological factors in paediatric allergic conditions.

The relationship between environmental exposure to chemicals and allergic diseases continues to be a highly debated phenomenon. The hygiene hypothesis suggests that cleaner environments lead to reduced exposure of children to infections and infestations, which subsequently leads to more allergies [2]. This is thought to be due to less antigenic stimulation of the childhood immune system leading to persistence of the T2 predominant immune activity which predisposes them to allergies [3]. Pesticides are also antimicrobial, so an environment with agrochemicals makes a “cleaner environment” which predisposes children to more allergies [4].

Pesticides and other agrochemicals are also considered irritants to the skin and respiratory system. Studies have shown that exposure is associated with precipitation of asthma in predisposed adults [5].

Pesticide aerosols or gases are known to stimulate functional irritant receptors in the airway promoting neurogenic inflammation leading to asthma. This can maintain chronic inflammation in the airways causing permanent damage to the bronchial epithelium [6].

There is well conducted research on environmental exposure and allergies in children. Though the power of these studies is not always adequate, some of them suggest that allergies in children are more common in environments exposed to pesticides [7]. On the other hand there are studies which show that environmental chemicals give rise to fewer allergies [8].

Sri Lanka is a low middle-income country and the world’s second largest tea exporter after Kenya. It is estimated that about 20% of the population is directly or indirectly involved with tea industry. Tea is grown in high, mid and low altitudes of the country and high grown tea estates are of relatively large extent compared to the other two types. They consist of about 27% of the total extent of tea grown land mass. Tea estate labourers in these large tea estates are provided housing in the same estate and usually a few generations of family members live in the same house. They are allowed to live and grow on their land but the ownership is with the estate they work in. There is very close contact of the family members with the agricultural land.

There are two types of agricultural systems: conventional agricultural systems and organic agricultural systems. In organic systems, the use of synthetic material is prohibited and in the conventional systems, the use of synthetic fungicides, insecticides, weedicides and foliar fertilizers are allowed. The conventional agriculture system of tea includes spraying of weedicides, fungicides and pesticides and use of chemical fertilizers. A significant proportion of the chemical drifts get into the environment, contaminating the air, water and the soil. Children get exposed to the effects of agrochemicals and fertilizers through soil, water and air more readily compared to the others due to their exploratory behaviour.

The objective of the current study was to compare the prevalence of allergic diseases among preschool children living in environments exposed to agrochemicals and artificial fertilizers under conventional agriculture, with those living with environmentally friendly cultivation practices under organic cultivation.

## Methods

### Study area

The study was conducted in Idalgashinna Bio tea project in Haputale, which is using only organic compounds and St Coombs estate Thalawakelle, which utilizes conventional agricultural practices. The former estate has not used artificial chemicals or fertilizer for the last 25 years and is a premier certified organic tea producer of the country. Both estates are located in high altitudes of the country with similar weather patterns.

Both of the estates are involved in producing high grown tea. The work force lives in a similar environment. The resident workers of both estates are of South Indian origin. They have been brought to the country by British rulers in the colonial era and have been living and working in the estates since. They are amply provided with general infrastructure facilities and medical aid in both systems within the estates. The workers and their families closely associate themselves with the tea plantations due to close proximity and for their daily needs.

The study was conducted during a 6-month period, as a survey using a self-administered questionnaire followed by clinical evaluation by medical officers. Informed written consent was obtained from the parents and assistance was provided by trained research assistants to complete the questionnaire. They were shown a video clip of wheezing, allergic rhinitis and pictures of eczema. All children between the ages of 1 year and 5 years living in St Coombs tea estate, Thalawakelle (conventional estate) and Idalgashinna Bio tea project, Haldummulla, Haputale (organic estate) were recruited to the study. The questionnaire used was a modified International Study of Asthma and allergies questionnaire (ISAAC) for 6–7 years which was adapted, translated to Tamil and pretested for validity by the preschool population used. It contained sections on preschool wheezing, eczema and allergic rhinitis. Those children screened with likely symptoms were later evaluated by three medical officers experienced in childhood diseases. The cases in which the diagnosis was uncertain to the medical officers were assessed by a consultant paediatrician. The data was entered into excel sheet and analysed by t-test for possible associations in the two systems. Ethical approval for the study was obtained from the institutional ethical review committee, Faculty of Medicine, University of Peradeniya.

## Results

A total of 81 preschool children in the Idalgashinna Bio tea project, Haldummulla, Haputale and 101 preschool children in the St Coombs estate Thalawakelle were evaluated out of 118 and 120 eligible children in the two estates, respectively. One child from Haputale estate was excluded from the study because of having cerebral palsy (Table 1).

**Table 1:** Demographic data of preschool children in St Coombs Estate Thalawakelle and Idalgashinna Bio Tea Project

	St. Coomb’s Estate, Thalawakelle	Idalgashinna Bio Tea Project, Haldummulla, Haputale
Total eligible	120	118
Total further evaluated	101	81
Boys	53	41
Girls	48	40

### Preschool wheezing, allergic rhinitis, eczema

In the organic estate (Haputale), 28 (34%) children had confirmed recurrent wheezing. In Thalawakelle estate the corresponding percentage was 59.8%.(p < 0.000) (Table 2).

**Table 2:** Allergic diseases of the preschool children in St. Coombs Estate Thalawakelle and Idalgashinna Bio Tea Project

	St. Coombs Estate, Thalawakelle			Idalgashinna Bio tea project, Haldummulla, Haputale			P value	Significant difference
Total	120			118				
Total further evaluated	101			81				
		**Total**	**%**		**Total**	**%**		
Wheezing	49	101	48.51	28	81	34.56	< 0.0001	Yes
Eczema	14	101	13.86	11	81	13.58	0.083	No
Allergic Rhinitis	33	101	32.67	13	81	16.04	< 0.0001	Yes
Any allergy	76	101	75.24	40	81	49.38	< 0.0001	Yes

In the two estates 3/101 and 13/81 respectively, had allergic rhinitis. The children at organic (Haputale) estate had a significantly lower prevalence of the condition compared to their conventional estate (Thalawakelle) counterparts. The least common allergic manifestation in both groups was eczema. There was no statistical difference in the number of patients with eczema in the two groups.

### Eosinophilia

Blood counts were done in 65 and 44 subjects consenting in Haputale and Thalawakelle estates respectively. The eosinophilia was more common in the Thalawakelle children (15/44) than the Haputale children (17/65), but the difference was not statistically significant (p = 0.157).

### Any allergy

Having any kind of allergic condition, including wheezing, eczema and allergic rhinitis at any point of their lives were analysed. Forty out of 81 respondents in Haputale estate and 76 out of 101 in Thalawakelle estates had at least one type of allergic condition. This shows that the Thalawakelle children had significantly higher prevalence of allergic conditions (p < 0.0001).

### Risk factors for allergy

Out of the subjects who have had any type of allergic conditions, the number of subjects who were breast fed for more than 18 months was calculated. Sixteen subjects in Haputale (40%) and 27 subjects in Thalawakelle (35.5%) had such history. The result was *not* significant.

Out of those with any type of allergy, the proportion of those exposed to paternal smoking in Haputale was 0.40. The proportion in Thalawakelle was 0.42. This was also not statistically significant.

## Discussion

Exposure to agricultural chemicals and chemical fertilizers can have a severe impact on health. According to a study done in Lebanon, any exposure to pesticides, including residential, para-occupational and domestic, was associated with respiratory disease and chronic respiratory symptoms [9].

Children living in agricultural lands can get exposed to chemicals and toxins by their natural urge to taste substances, having meals without proper hand washing, drift of sprayed chemicals, contact with chemical exposed surfaces, breast feeding by mothers contaminated by chemicals during work, etc. Studies have shown that chronic exposure to organophosphates can lead to neurodevelopmental and growth problems in children [10]. Environmental exposure and allergic diseases has been a topic of many studies with varying findings.

Our study was conducted in a unique setting where there is very close contact of the children with the agricultural land they live in. The prevalence of the common allergic conditions was evaluated in high grown tea estates—one estate using conventional methods and the other using only organic methods of farming. The latter estate has not used any type of artificial additives in the environment for 25 years.

The study revealed that there was a significant difference in prevalence of allergic conditions in the children in the conventional estate exposed to agrochemicals when compared with children of the organic estate. This could be due to the “cleaner” environment of the conventional estate due to the use of agrochemicals which are deterrents to the growth of microorganisms.

The phase 3 trial of ISAAC study showed that 27% of Sri Lankan children of the 6- to 7-year age group had wheezing over the preceding 12 months [11]. There are no published data regarding prevalence of preschool wheezing in Sri Lanka. Children of both estates showed a higher prevalence of recurrent wheezing which follows the recognised higher prevalence in preschool children compared to older children. Studies done in other countries show the fact that all preschool wheezing disorders have increased over the years and it is probable that factors unrelated to atopy are implicated in the changing epidemiology of wheeze in childhood [12]. It is well explained that, in pre-school children, it is not easy to differentiate allergic wheezing conditions from non allergic wheezing as in older children [13]. Therefore we did not attempt differentiate the different phenotypes of preschool wheezing in our study.

The prevalence of wheezing as well as allergic rhinitis was also significantly higher in the conventional estate. Similarly, the prevalence of children with allergic rhinitis was significantly higher in the children living in conventional estate, though prevalence of eczema had no significant difference between the two estates. Due to the lower prevalence of eczema, a bigger sample size is necessary to evaluate this further.

The full blood counts were done on the subjects, and eosinophilia was analysed, as an indicator of allergy. Though the proportion of subjects with eosinophilia was higher in Thalawakelle, which is using conventional methods, than in Haputale, the difference was not statistically significant. As eosinophilia can occur due to many other factors, this may not be entirely allergy related.

## Conclusions

This study highlights an important health impact when living in an environment using agrochemicals and artificial fertilizers. The study showed that both the populations had a higher prevalence of allergic diseases, but the children not exposed to agrochemicals showed significantly lower rates of allergic diseases.

Tea is a very important revenue earner to the country with a significant number of the population benefitting from this enterprise. It is also important to make sure the environment of the workers and their children are safe in order to maximise the economic benefit. The results of the study show that stricter rules and other measures are needed to minimize exposure of children to the harmful effects of these toxins.
